# Lives of Eminent American Physicians and Surgeons of the Nineteenth Century

**Published:** 1861-03

**Authors:** 


Lives of Eminent American Physicians and Surgeons of the Nineteenth Century. Edited by Samuel D. Gross, M. D., Professor of Surgery in Jefferson Medical College. Philadelphia: Lindsay & Blakiston, 1861.

This is a large octavo volume of 836 pages, published in elegant style. It contains pretty full and highly interesting biographical sketches of thirty-two eminent American physicians and Surgeons, written by almost as many different authors. We think no reader can look over the following, from the table of contents without feeling, a strong desire to possess the work.

Biography of Benjamin Rush, by Dr. Samuel Jackson,
" John Warren, by Dr. Buckmaster Brown,
" Caspar Wistar, by Dr. Caspar Morris.
" John Syng Dorsey, by Dr. Sam'l D. Gross.
" Samuel Bard, by Dr. James P. White.
" Ephraim McDowell, by Dr. Sam'l D. Gross.
" Samuel Brown, by Dr. LaRoche,
" John D. Godman, by Dr. T. G. Richardson.
" Samuel L. Mitchell, by Dr. John W. Francis.
" David Hosack, by Dr. Alex. E. Hosack,
" Phillip Syng Physick, by Dr. John Bell.
" John Eberle, by Dr. Thos. D. Mitchell,
" Wm. J. Macnevin, by Dr. John W. Francis.
" James Thatcher, by Dr. N. S. Davis,
" George McClellan, by Dr. J. H. B. McClellan.
" Jacob Randolph, Dr. J. Aitken Meigs,
" Amariah Brigham, by Dr. E. K. Hunt,
" Chas. A. Luzenberg, by Dr. Thos. M. Logan.
" Joseph Hartshorne, by Dr. E. Hartshorne,
" Sam'l Geo. Morton, by Dr. Sanford B. Hunt.
" John B. Beck, by Dr. C. R. Gilman,
" Daniel Drake, by Dr. Samuel D. Gross.
" Nathaniel Chapman, by Dr. J. B. Biddle,
" Lewis C. Beck, by Dr. Alden March,
" Wm. E. Horner, by Wm. Horner, Esq.,
" John A. Sweet, by Dr. Austin Flint,
" Elisha Bartlett, by Dr. Samuel H. Dickson,

Biography of Moreton Stille, by Dr. Sam'l H. Dickson,
" Theodric R. Beck, Dr. Frank H. Hamilton,
" John C. Warren, by Dr. Edward Warren,
" Charles Frick, by Dr. Frank Donaldson,

No work has issued from the medical press, in this country, that should meet with a more cordial reception, from all classes of readers. We hope the rapid sale of this, will induce the speedy publication of an additional volume.

For sale by S. C. Griggs & Co., of this city.

